# Longitudinal tumor hypoxia imaging with [^18^F]FAZA-PET provides early prediction of nanoliposomal irinotecan (nal-IRI) treatment activity

**DOI:** 10.1186/s13550-015-0135-x

**Published:** 2015-10-19

**Authors:** Jinzi Zheng, Stephan G. Klinz, Raquel De Souza, Jonathan Fitzgerald, David A. Jaffray

**Affiliations:** TECHNA Institute for the Advancement of Technology for Health, University Health Network, 101 College Street, Rm 7-302, Toronto, Ontario M5G 1L7 Canada; Department of Radiation Physics, Princess Margaret Cancer Centre, University Health Network, Toronto, Ontario Canada; Institute of Biomaterials & Biomedical Engineering, University of Toronto, Toronto, Ontario Canada; Merrimack Pharmaceuticals, Inc., Cambridge, MA USA; Radiation Oncology, University of Toronto, Toronto, Ontario Canada

**Keywords:** Hypoxia, FAZA, PET, Irinotecan, Liposome

## Abstract

**Background:**

Non-invasive measurement of tumor hypoxia has demonstrated potential for the evaluation of disease progression, as well as prediction and assessment of treatment outcome. [^18^F]fluoroazomycin arabinoside (FAZA) positron emission tomography (PET) has been identified as a robust method for quantification of hypoxia both preclinically and clinically. The goal of this investigation was to evaluate the feasibility and value of repeated FAZA-PET imaging to quantify hypoxia in tumors that received multi-dose chemotherapy.

**Methods:**

FAZA-PET imaging was conducted over a 21-day period in a mouse xenograft model of HT-29 human colorectal carcinoma, following multi-dose chemotherapy treatment with irinotecan (CPT-11) or nanoliposomal irinotecan (nal-IRI, MM-398).

**Results:**

Tumors treated with 10 mg/kg nal-IRI maintained significantly lower levels of hypoxia and smaller hypoxic fractions compared to tumors that received 50 mg/kg CPT-11. Specifically, differences in FAZA uptake were detectable 9 days before any significant differences in tumor volume were observed between the treatment groups.

**Conclusions:**

These findings highlight the potential use of FAZA-PET as an early marker of treatment response following multi-dose chemotherapy.

**Electronic supplementary material:**

The online version of this article (doi:10.1186/s13550-015-0135-x) contains supplementary material, which is available to authorized users.

## Background

Tumor hypoxia is strongly linked to aggressive disease progression and resistance to therapy [1]. Specifically, hypoxia-induced chemoresistance is associated with (1) reduced intratumoral perfusion, which hinders drug access to hypoxic areas; and (2) the quiescent state of hypoxic cells, which render DNA structure modifying chemo agents ineffective. The degree of hypoxia is a dynamic quantity that is influenced by physiological factors. It is therefore important to assess hypoxia in tumors before, during, and after therapy.

Advances in non-invasive imaging have resulted in the development and clinical exploration of a number of hypoxia targeted agents for positron emission tomography (PET), including [^18^F]fluoromisondazole (FMISO) [2, 3], [^18^F]fluoroazomycin arabinoside (FAZA) [4–11], [^64^Cu]diacetyl-bis(N4-methylthiosemicarbazone (ATSM) [12–14], and [^18^F]flortanidazole (HX4) [15–19]. Recent reports have shown that [^18^F]FAZA may offer superior sensitivity in the detection of hypoxic regions due to faster systemic clearance from non-hypoxic tissues and, therefore, lower non-specific background activity compared to [^18^F]FMISO [20]. In addition, preclinical validation has demonstrated a good agreement between intratumoral FAZA uptake, Eppendorf electrode measurements, and pimonidazole staining [5], while [^64^Cu]ATSM failed to show good correlation with carbonic anhydrase IX (CAIX) immunostaining [21]. Furthermore, FAZA-based PET quantification of hypoxia proved to be highly reproducible in untreated animals when imaged 24 h apart [6]. Both preclinical and clinical reports have shown encouraging prognostic and predictive power of FAZA-PET-based hypoxia imaging, particularly when used in conjunction with radiotherapy [4, 8, 9]. These findings support the employment of FAZA-PET as an effective imaging technique to quantify hypoxia. Although other PET-based tracers such as [^18^F]-fluorodeoxygluocose (FDG) and [^18^F]-fluorothymidine (FLT) have shown potential in predicting early treatment response in patients and animal models of cancer [22, 23], they do not directly provide information on the hypoxia status of a tumor.

Nanoliposomal irinotecan (nal-IRI, MM-398) is a highly stable liposomal nanocarrier formulation of irinotecan hydrochloride (CPT-11) that significantly prolongs the pharmacokinetics and tumor bio-distribution of the free drug [24, 25]. Nal-IRI greatly increases the duration of the therapeutically active metabolite, SN-38, within tumors, which becomes a better correlate to in vivo activity of either free or nanoliposomal irinotecan than SN-38 exposure when measured as the area-under-the-curve (AUC) [24]. Nal-IRI has shown activity in a number of preclinical tumor models [24, 25] and has met its clinical endpoint in a phase III clinical trial in gemcitabine-refractory pancreatic cancer [26], a tumor indication that is characterized by low vascular density as well as numerous and severe hypoxic regions [27].

We and others have observed that sustained exposure as provided by nanoformulations of irinotecan can reduce levels of hypoxia or hypoxia-regulated protein markers relative to untreated tumors after either prolonged treatment [28] or as immediate as following a single-dose administration. However, such endpoint assessments do not provide a comprehensive description of the dynamic changes in tumor hypoxia characteristics during exposure to a course of irinotecan-based chemotherapy, and no investigation to date has explored the feasibility and performance of hypoxia imaging for quantification of acute (i.e., hours) and chronic (i.e., days) hypoxia changes in such a setting. Here, we report the use of FAZA-PET for repeated and longitudinal monitoring of tumor hypoxia changes in a mouse xenograft model of HT-29 colorectal cancer before, during, and after three weekly chemotherapy administrations of either free irinotecan or nal-IRI.

## Methods

### Animal model

Studies were approved by the University Health Network Animal Care Committee and adhered to the ethical guidelines of the Canadian Council on Animal Care. Female, 6- to 8-week-old NOD/SCID mice (Ontario Cancer Institute, Toronto, Canada) were inoculated subcutaneously with 1 × 10^7^ HT-29 human colorectal adenocarcinoma cells (ATCC, Manassas, VA, USA), in a 100 μL injection volume, at both dorsal flank sites such that each mouse bore bilateral tumors. HT-29 cells represent a goblet-like subtype of colorectal adenocarcinoma [29]. Tumor growth was monitored using caliper-based measurements. Studies began 17 days post-inoculation, when tumors reached a mean volume of 307.5 ± 131.7 mm^3^ (15 mice, 30 tumors).

### Chemotherapy treatment

Animals (*n* = 15) were randomized into three treatment groups: (1) irinotecan hydrochloride (referred to as irinotecan hereafter) administered at 50 mg/kg, (2) nal-IRI at 5 mg/kg, and (3) nal-IRI at 10 mg/kg. Based on a mechanistic pharmacokinetic model, 10 mg/kg of nal-IRI or 50 mg/kg irinotecan was estimated to result in similar AUC exposure to SN-38 in both plasma and tumor, while 5 mg/kg nal-IRI and 50 mg/kg irinotecan showed a comparable duration of SN-38 levels above a critical intratumoral threshold of 120 nmol/L [24]. All treatment doses were known to suboptimally control tumor growth in the bilateral subcutaneous HT-29 tumor model. Each treatment group was composed of a total of 10 tumors (five animals bearing two tumors each). A total of four weekly administrations were given *i.v.* on day 0, 7, 15, and 21.

### In vivo imaging

**Fig. 1:**
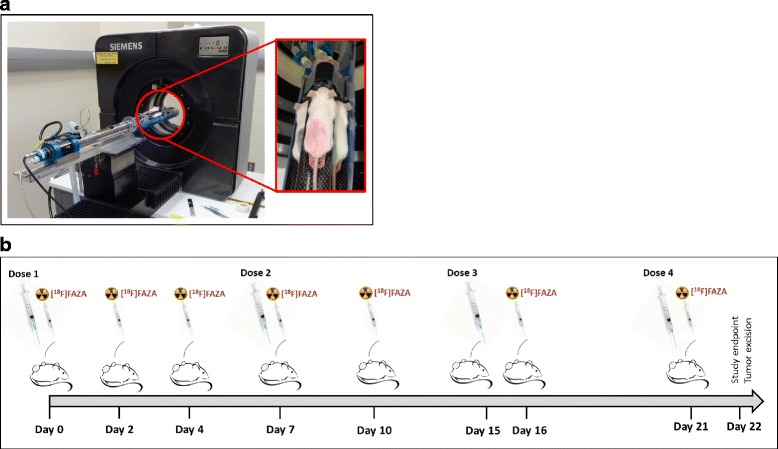
Illustration of **a** the triple mouse PET imaging set-up and **b** the experimental workflow. Day 0 corresponds to the day of treatment initiation. Note that the fur of the mice was colored for identification purposes

A total of seven FAZA-PET/CT imaging sessions were performed on day 0, 2, 4, 7, 10, 16, and 21 following initiation of treatment using a triple mouse imaging bed (Fig. 1). [^18^F]FAZA was produced by CanProbe (Ontario, Canada) with a radiochemical purity of 95.7 ± 3.7 % (calculated over seven productions). PET imaging (Focus 220, Siemens) was performed at 2-h post-FAZA administration (0.79 ± 0.06 MBq/g of body weight, Additional file 1: Figure S1). Imaging at 2-h post-FAZA injection was reported to be a desirable imaging time point based on tracer kinetics at the tumor site (i.e., reaching steady state) and on the fact that at this time, post-injection, the tumor tracer uptake levels correlated well with tissue hypoxia [5, 30]. Each PET acquisition consisted of a 20-min emission scan followed by an 8-min ^57^Co transmission scan for attenuation and scatter correction. Then, a CT scan (GE Locus Ultra, 80 kVp, 50 mA) was performed with animals in the same position in order to provide anatomical data for image registration. Treatment response was quantified based on CT tumor volumes.

### Image analysis

The PET and CT datasets were registered, contoured, and analyzed using the Inveon Research Workplace software (IRW 4.0, Siemens). The hypoxic fraction is defined as the number of tumor voxels with FAZA-PET signal values above a set threshold over the total number of tumor voxels. The hypoxia signal threshold value was defined as the mean FAZA-PET signal value measured in the upper leg muscle of the same mouse + 3 standard deviations [31].

### Histology

Tumor-specific hypoxia status at the study endpoint (day 22) was confirmed by immunohistochemistry. Animals received an intraperitoneal administration of EF5 (0.1 mM EF5/g body weight) 2 h before euthanasia, and portions of the excised tumors were fixed, sectioned, and stained for hematoxylin and eosin (H&E), EF5 (anti-EF5 ELK3-51), and CAIX (anti-CAIX M75). Image acquisition was done with an Aperio Scanscope AT. Analysis of the histology images were performed using Definiens Tissue Studio (Definiens AG, Munich, Germany).

### Statistical analysis

Differences between means for the different treatment groups were compared using one-way ANOVA or an independent samples *t* test where equal variances are not assumed and with a confidence interval of 95 %. Differences between means for the same treatment group on different days were compared using a paired-samples *t* test with a confidence interval of 95 %. All statistical calculations were performed using SPSS version 22 (IBM, Armonk, NY, USA).

## Results and discussion

### Treatment group randomization and baseline FAZA uptake

**Fig. 2:**
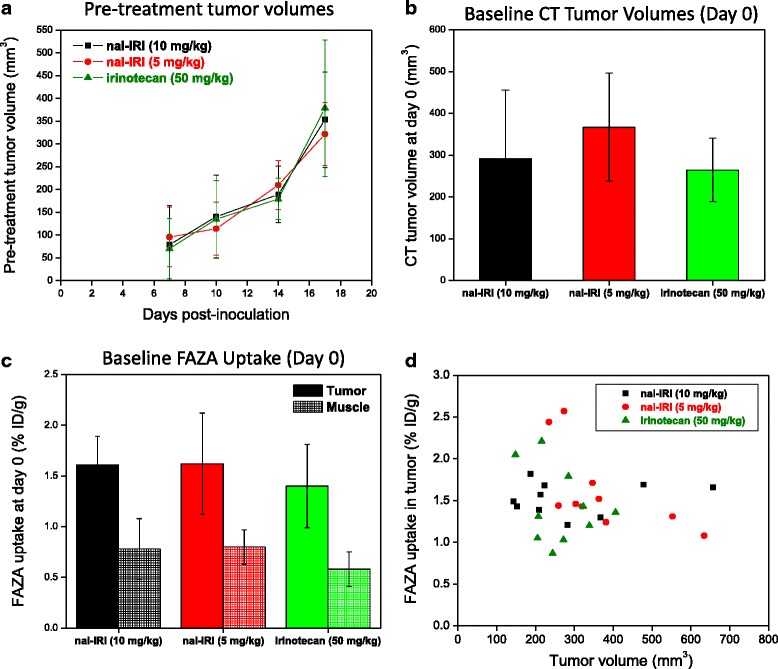
Study baseline data including **a** pre-treatment mean tumor growth kinetics for each group (day 17 post-inoculation corresponds to day 0 of treatment initiation), **b** the baseline tumor volume obtained by contouring the volumetric CT data sets (*p* = 0.202), **c** the pre-treatment tumor (*p* = 0.579) and muscle (*p* = 0.304) FAZA uptake, and **d** the lack of correlation between tumor FAZA uptake and tumor volume. Each treatment group is composed of 5 mice, each bearing bilateral subcutaneously implanted HT-29 colon cancer xenograft, for a total of 10 tumors per treatment group

In order to ensure that the response is consistent across animals and tumors that received the same treatment, mice bearing bilateral tumors were randomized into three treatment groups (irinotecan at 50 mg/kg, nal-IRI at 5 mg/kg, and nal-IRI at 10 mg/kg) based on pre-treatment growth kinetics (Fig. 2a) and caliper-measured tumor volume at treatment day 0 (Fig. 2b). Figure 2a shows no significant difference in the tumor growth curves from day 7 to day 17 post-tumor inoculation. Figure 2b illustrates the mean CT tumor volumes for each group (a total of 10 tumors in 5 mice per group), assessed after animals were already randomized on the day of treatment initiation. No statistically significant difference was found among groups (*p* = 0.202 for CT-measured tumor volumes, *p* = 0.539 for caliper-measured tumor volumes). The correlation (*R*^2^ = 0.904, Additional file 1: Figure S2) between the tumor volumes estimated based on digital caliper measurements and CT volume contours was within a 10 % tolerance (caliper volume = 0.938 × CT volume, 30 tumors over 7 measurement days).

On the day of treatment initiation, baseline FAZA uptake in mean %ID/g for both tumor and muscle (ROI drawn on the inner thigh muscle) was calculated. No statistically significant differences between the baseline tumor uptake for the three groups was found (*p* = 0.579, tumor uptake = 1.40 ± 0.41 to 1.62 ± 0.50 %ID/g, Fig. 2c). Most importantly, no correlation (*R*^2^ = 0.008, Fig. 2d) was found between the mean FAZA tumor uptake and the CT tumor volume at study baseline (on the day of treatment initiation). Muscle FAZA uptake (Additional file 1: Figure S3) averaged ~50 % of the uptake measured in tumors at baseline consistent with previous publications [4, 8]. The differences measured in the baseline mean FAZA muscle uptake among the three treatment groups were relatively small (0.58 ± 0.18 %ID/g for the irinotecan group, 0.78 ± 0.32 %ID/g for the 5 mg/kg nal-IRI group, and 0.80 ± 0.18 %ID/g for the 10 mg/kg nal-IRI group) and were not statistically significant (*p* = 0.304). Muscle FAZA uptake was not used to normalize FAZA tumor uptake (i.e., to calculate tumor-to-muscle ratios) at the various imaging time points, since a reproducibility study previously conducted by our group [32] had shown that there is higher day-to-day variation in the muscle FAZA uptake compared to tumor.

### Treatment time course FAZA uptake

**Fig. 3:**
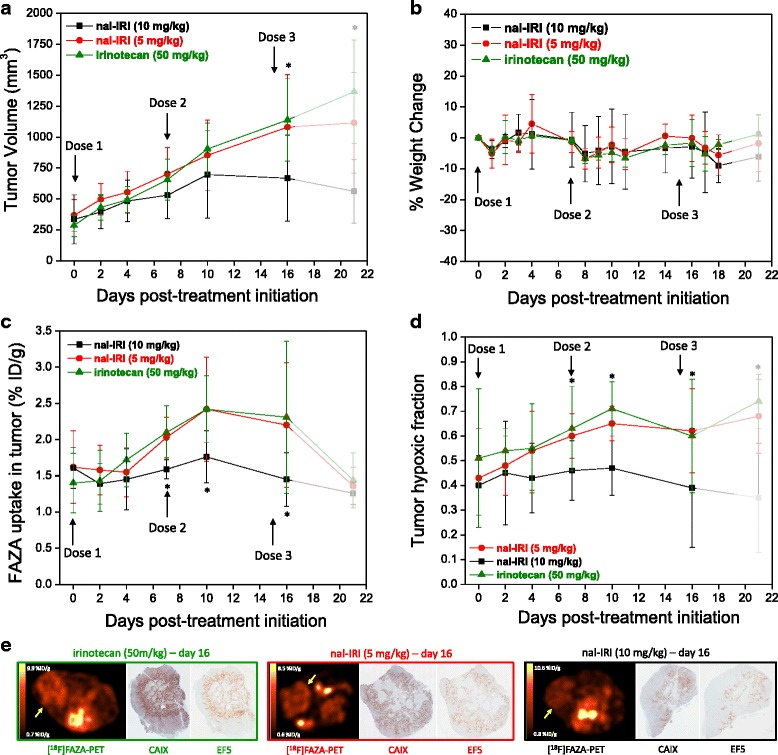
**a** Tumor volume changes and **b** animal weight fluctuations over the study period of 21 days. **c** Longitudinal tumor FAZA uptake (%ID/g) and **d** tumor hypoxic fraction calculated from the PET data set. Each data point (except for day 21, transparently masked in *gray*) represents the mean ± standard deviation obtained from five animals, each bearing bilateral tumors, for a total of 10 tumors per treatment group. *Asterisk* denotes statistically significant difference (*p* < 0.05) between the nal-IRI (10 mg/kg) and either one or both the other two treatment groups. **e** Three sets of representative PET image (left) and CAIX (middle) and EF5 (right) stained tumor sections obtained from three animals, one belonging to each treatment group. Qualitative demonstration that the degree of FAZA-PET signal is confirmed by the level of CAIX and EF5 immunohistochemistry staining (i.e., high FAZA uptake correlates to high CAIX and high EF5 staining)

Response to treatment was evaluated over a 3-week period through CT-based tumor volume measurement (Fig. 3a). The seven imaging sessions and the three treatment doses were well-tolerated by all animals (Fig. 3b). The mean FAZA uptake measured in the 30 tumors (15 mice) ranged between 0.83 and 4.29 %ID/g over the course of the study (Fig. 3c). Treatment with 10 mg/kg nal-IRI maintained significantly lower levels of hypoxia and smaller hypoxic fractions compared to tumors that received irinotecan (Fig. 3c, d), and these differences between the treatment groups were significant as early as day 7 post-treatment initiation (Table 1). Tumor growth control was also apparent in the 10 mg/kg nal-IRI treatment group (Fig. 3a). However, differences in tumor volume only became statistically significant on day 10 (nal-IRI at 10 mg/kg vs. irinotecan at 50 mg/kg, *p* = 0.038) and day 16 (nal-IRI at 10 mg/kg vs. irinotecan at 50 mg/kg, *p* = 0.006; nal-IRI at 10 mg/kg vs. nal-IRI at 5 mg/kg, *p* = 0.029) (Table 1). This suggests that tumor hypoxia imaging using FAZA-PET has the potential to provide early prediction of treatment response. Five animals (one from each of the nal-IRI groups and three from the irinotecan treatment group) were sacrificed between day 16 and 21 for ethical reasons (tumor ≥ 1.5 cm in any direction). As a result, the data reported for day 21 only represents the surviving subgroup and is therefore skewed toward better response as shown by an overall decrease in tumor FAZA uptake (Fig. 3c). It is interesting to note that the tumor hypoxic fraction measure (Fig. 3d) better correlates with tumor response on day 21 (Fig. 3a) than the mean FAZA tumor uptake (Fig. 3c). This suggests that quantification of tumor hypoxia using the hypoxic fraction measure may be a more reliable evaluation metrics than the mean FAZA uptake value.

**Table 1:**
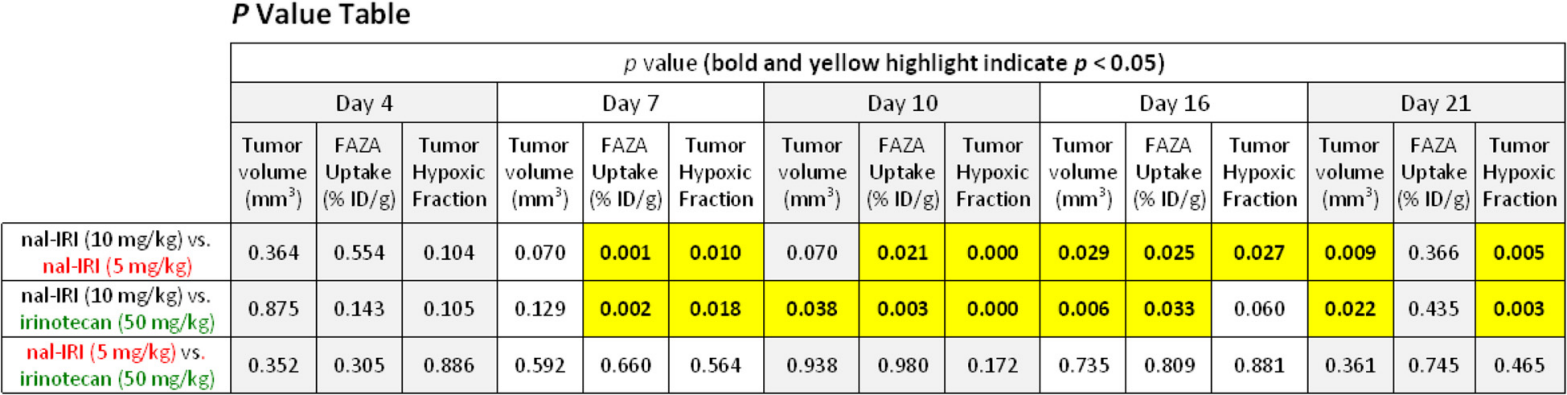
*P* values calculated from independent samples *t* tests (two-tailed, equal variance not assumed) for tumor volume, tumor FAZA uptake, and tumor hypoxic fraction parameters at various study time points.

Statistically significant difference in tumor FAZA uptake (expressed both in terms of %ID/g and tumor hypoxic fraction) is found on day 7 post-treatment initiation between the nal-IRI (10 mg/kg) and the other two treatment groups. Statistically significant difference in tumor volume, for the same groups, is seen on day 10 and 16. A total of 10 tumors were included in the analysis for each group on days 4 through 16. On day 21, the number of tumors per group was 4, 6, and 8 for irinotecan, nal-IRI (10 mg/kg) and nal-IRI (5 mg/kg), respectively

**Fig. 4:**
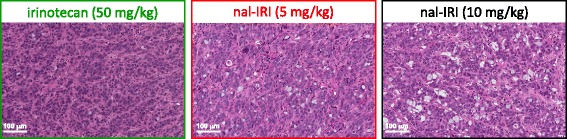
Representative H&E stained tumor sections (20× magnification, day 16) showing a decrease in cellular density following treatment with nal-IRI at a dose of 10 mg/kg compared to treatment with nal-IRI at 5 mg/kg and treatment with irinotecan at 50 mg/kg

Initial histological validation of the FAZA-PET signal was done by staining with two distinct hypoxia markers, CAIX and EF5, on adjacent tumor tissue slides (Fig. 3e). The degree of FAZA-PET tumor uptake is proportional to the amount of CAIX and EF5 positive staining. Specifically, the percent CAIX and EF5 positive tumor areas for the 10 mg/kg nal-IRI group (17.6 ± 7.4 % and 1.9 ± 1.6 %, respectively) are significantly lower (*p* < 0.05) compared to those treated with 5 mg/kg of nal-IRI (28.8 ± 8.5 % and 5.8 ± 3.9 %, respectively) and 50 mg/kg of irinotecan (29.2 ± 5.5 % and 9.2 ± 4.7 %, respectively) This is in agreement with the significantly lower FAZA-positive tumor fraction quantified using the PET data (Table 1). A decrease in cellular density following treatment with nal-IRI at 10 mg/kg compared to the other treatments was also observed (Fig. 4).

### Longitudinal changes in tumor FAZA uptake

**Fig. 5:**
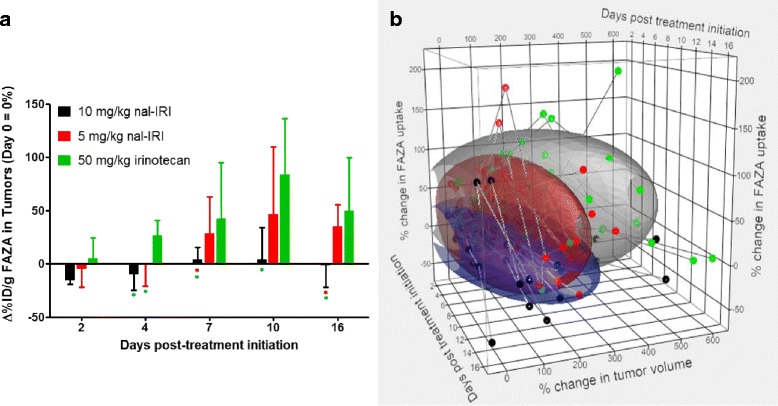
**a** Longitudinal relative changes in tumor FAZA uptake with respect to pre-treatment values (%ID/g on day 0). Median values are plotted with the interquartile range indicated by bars. *Green asterisk* and *red asterisk* indicate statistically significant difference with respect to the 50 mg/kg irinotecan group and the 5 mg/kg nal-IRI group (*p* < 0.05), respectively. **b** Three-dimensional scatterplot of individual tumors at each treatment day with regard to % change in FAZA uptake from baseline (day 0) and % tumor volume change from day 0. For each treatment group, contour ellipsoids (ellipsoid coverage of data points = 70 %) are presented. Tumors are treated with 10 mg/kg nal-IRI (blue ellipsoid), 5 mg/kg nal-IRI (red ellipsoid), and 50 mg/kg irinotecan (gray ellipsoid)

Relative changes in tumor volume and hypoxia over time with respect to baseline values (day 0) were calculated for each tumor individually. The relative change in FAZA uptake in tumors over the 16-day period compared to baseline is shown in Fig. 5a. An increase in median FAZA uptake can be observed for the group that received 50 mg/kg of irinotecan starting as early as 4-day post-treatment initiation and for the group that received 5 mg/kg of nal-IRI 7-day post-treatment initiation. Only with the nal-IRI treatment at 10 mg/kg did the tumors maintain their pre-treatment hypoxia level on day 7 (*p* = 0.264 with respect to day 0 baseline values, paired-samples *t* test) and beyond following treatment initiation, despite significant increases in their tumor volumes (*p* < 0.005 for day 7, volumes with respect to day 0 baseline, paired-samples *t* test). Thus, the treatment with 10 mg/kg nal-IRI was able to stabilize the hypoxia levels in tumors. In addition, disease progression in terms of tumor volume increase was better controlled in this treatment group compared to the other two treatment groups (Fig. 3a).

Normalized time course trajectories for individual tumors of changes in FAZA uptake and tumor volume with respect to baseline values are shown in a three-dimensional plot (Fig. 5b). Ellipsoidal contours were generated for all trajectory data points from each treatment group (ellipsoid coverage = 70 % of data points). We observed treatment-specific divergence among all three treatment groups; data points from tumors treated with 10 mg/kg nal-IRI were clustered around the smallest changes in both tumor volume and FAZA uptake (blue ellipsoid), while data points from tumors treated with 50 mg/kg irinotecan were clustered around the largest changes (gray ellipsoid). The data points from tumors treated with 5 mg/kg nal-IRI (red ellipsoid) were shifted toward intermediate changes, particularly along the percent change in FAZA uptake axis. In addition, the trajectory path lengths of tumors treated with nal-IRI at either 5 or 10 mg/kg were significantly different (*p* = 0.0012, one-way ANOVA) from those treated with free irinotecan (data not shown). This time course assessment of individual tumor performance allowed for identification of more subtle differences that was not appreciated using group statistics alone.

## Discussion

Longitudinal imaging studies are challenging to perform, even in well-controlled preclinical animal models. In hypoxia imaging, the baseline hypoxia status of the tumor can be significantly different even for tumors originating from the same cell line with similar volumes and growth rates. Considerable effort was thus taken to minimize randomization bias as this could become more pronounced over the course of repeated measurements performed in the same animals and tumors. Our study showed the feasibility of performing FAZA-PET imaging for monitoring response to treatment, even in a relatively heterogeneous group of tumors with higher variability in pre-treatment tumor volume and hypoxia level, when appropriate randomization steps are employed. Using these methods, this study further demonstrated that FAZA-PET imaging-based tumor hypoxia assessment may be utilized as an early predictor of chemotherapy treatment response. Specifically, our findings illustrate that differences in tumor hypoxia can be detected significantly earlier (3 to 9 days) than tumor volume differences between treatment groups.

Our results show no immediate hypoxia reduction, both in terms of %ID/g of FAZA tumor uptake and tumor hypoxic fraction, on days 2 to 4 after treatment with free irinotecan or nal-IRI. This is consistent with the mechanism of action of camptothecins, which requires a prolonged exposure time to SN-38 for maximum cytotoxic effects [33]. Free irinotecan is rapidly cleared from plasma and tumor tissue, thereby not allowing sufficient time for tumor cells to be exposed to SN-38. Only with the nal-IRI treatment at 10 mg/kg did the tumors maintain their pre-treatment hypoxia level on day 7 and beyond following treatment initiation, despite significant initial increases in their tumor volumes, thus suggesting changes in the tumor microenvironment. Previous experiments in an HT-29 tumor model grown in NOD-SCID mice have shown that the 10 mg/kg nal-IRI dose achieves a prolonged SN-38 tumor duration of ~96 h, while the exposure with 50 mg/kg free irinotecan is ~36 h [24]. Similar to the published report on another liposomal irinotecan formulation, irinophore C [28], data from this study showed that tumors presented a less hypoxic profile following the 10 mg/kg nal-IRI treatment, and the prolonged SN-38 exposure resulted in decreased cellular density thus potentially alleviating solid stress and reducing blood vessel compression during tumor growth [34]. It has been shown that irinotecan and other camptothecins can inhibit the hypoxia-inducible factor-1α (HIF-1α) protein accumulation in vivo. This can interfere with the capacity of tumor cells to adapt to a hypoxic environment [10] and may increase their treatment sensitivity [1]. Furthermore, the modulation of HIF-1α protein levels has been reported to occur independently of significant changes in intratumoral hypoxia [10]. However, this study was aimed at quantifying longitudinal tumor hypoxia changes and could not address these mechanistic questions.

Results from our investigation confirmed that a dose of 10 mg/kg of nal-IRI was sufficient to provide growth control in the bilateral HT-29 subcutaneous xenograft model, and it demonstrated a significant benefit in therapeutic response compared to the other two treatment groups. The administration of a therapeutically more effective dose of nal-IRI (i.e., 20 mg/kg), while safe and efficacious [35], would have reduced sensitivity in the measurement of FAZA uptake due to potential disappearance of tumor hypoxia following partial or complete response and could also have introduced measurement bias due to rapid tumor volume shrinkage. Dosing in murine models for both nal-IRI and free irinotecan employed in this study scales appropriately to the clinical setting where nal-IRI is given at a dose density of 40 mg/m^2^/week [36].

The apparent discordance between FAZA uptake (Fig. 3c) and hypoxic fraction (Fig. 3d) on day 21 post-treatment initiation for the nal-IRI treated groups is likely due to a decrease in focal areas of intense hypoxia which diminishes the mean tumor FAZA uptake value, while the overall volume fraction of hypoxia is maintained in the tumor compared to day 16. This observation motivates further studies aimed at investigating the intratumoral hypoxia heterogeneity. In fact, more sophisticated image analysis and acquisition strategies exist to further increase the sensitivity of FAZA-PET for hypoxia measurement. For example, quantification of intratumoral heterogeneity can be performed both in terms of subregional steady-state FAZA uptake and variations in distribution, as well as uptake and clearance kinetics through the identification of intratumoral multi-voxel clusters. In addition, kinetic modeling of dynamic FAZA uptake enables improved inter-tumor normalization achieved through the measurement of the perfusion characteristics of each tumor during the early imaging frames.

## Conclusions

This study demonstrated the feasibility of performing longitudinal and repeated tumor hypoxia assessment using FAZA-PET imaging for early prediction of treatment response. Statistically significant differences in hypoxia within tumor-size matched groups in response to different treatments were successfully detected. Specifically, the liposomal irinotecan formulation nal-IRI showed enhanced ability to halt progression of tumor hypoxia compared to free irinotecan. Overall, hypoxia changes following anti-cancer therapy has the potential to provide an early assessment of treatment activity.

## Additional file

Additional file 1:
**Figures S1–S3.** Figure S1. Mean FAZA administration dose (MBq/g) calculated for animals belonging to each treatment group over 7 imaging sessions. Figure S2. Tumor volume correlation between measurements performed using caliper and CT. Figure S3. Longitudinal muscle FAZA uptake (%ID/g) measured from the CT data set over a 21-day period following the first treatment dose administration (day 0). Each data point (except for day 21, transparently masked in gray) represents the mean ± standard deviation obtained from 5 animals. (PDF 86 kb)

## References

[1] Tredan O, Galmarini CM, Patel K, Tannock IF (2007). Drug resistance and the solid tumor microenvironment. J Natl Cancer Inst.

[2] Hendrickson K, Phillips M, Smith W, Peterson L, Krohn K, Rajendran J (2011). Hypoxia imaging with [F-18] FMISO-PET in head and neck cancer: potential for guiding intensity modulated radiation therapy in overcoming hypoxia-induced treatment resistance. Radiother Oncol.

[3] Lin Z, Mechalakos J, Nehmeh S, Schoder H, Lee N, Humm J (2008). The influence of changes in tumor hypoxia on dose-painting treatment plans based on 18 F-FMISO positron emission tomography. Int J Radiat Oncol Biol Phys.

[4] Beck R, Roper B, Carlsen JM, Huisman MC, Lebschi JA, Andratschke N (2007). Pretreatment 18 F-FAZA PET predicts success of hypoxia-directed radiochemotherapy using tirapazamine. J Nucl Med.

[5] Busk M, Horsman MR, Jakobsen S, Keiding S, van der Kogel AJ, Bussink J (2008). Imaging hypoxia in xenografted and murine tumors with 18 F-fluoroazomycin arabinoside: a comparative study involving microPET, autoradiography, PO2-polarography, and fluorescence microscopy. Int J Radiat Oncol Biol Phys.

[6] Busk M, Mortensen LS, Nordsmark M, Overgaard J, Jakobsen S, Hansen KV (2012). PET hypoxia imaging with FAZA: reproducibility at baseline and during fractionated radiotherapy in tumour-bearing mice. Eur J Nucl Med Mol Imaging.

[7] Halmos GB, de Bruin LB, Langendijk JA, van der Laan BF, Pruim J, Steenbakkers RJ (2014). Head and neck tumor hypoxia imaging by 18 F-fluoroazomycin-arabinoside (18 F-FAZA)-PET: a review. Clin Nucl Med.

[8] Mortensen LS, Busk M, Nordsmark M, Jakobsen S, Theil J, Overgaard J (2011). Accessing radiation response using hypoxia PET imaging and oxygen sensitive electrodes: a preclinical study. Radiother Oncol.

[9] Schuetz M, Schmid MP, Potter R, Kommata S, Georg D, Lukic D (2010). Evaluating repetitive 18 F-fluoroazomycin-arabinoside (18FAZA) PET in the setting of MRI guided adaptive radiotherapy in cervical cancer. Acta Oncol.

[10] Guerin E, Raffelsberger W, Pencreach E, Maier A, Neuville A, Schneider A (2012). In vivo topoisomerase I inhibition attenuates the expression of hypoxia-inducible factor 1alpha target genes and decreases tumor angiogenesis. Mol Med.

[11] Chapman DW, Jans HS, Ma I, Mercer JR, Wiebe LI, Wuest M (2015). Detecting functional changes with [(18)F]FAZA in a renal cell carcinoma mouse model following sunitinib therapy. EJNMMI Res.

[12] Ballegeer EA, Madrill NJ, Berger KL, Agnew DW, McNiel EA (2013). Evaluation of hypoxia in a feline model of head and neck cancer using (64)Cu-ATSM positron emission tomography/computed tomography. BMC Cancer.

[13] Vavere AL, Lewis JS (2007). Cu-ATSM: a radiopharmaceutical for the PET imaging of hypoxia. Dalton Trans.

[14] Zhang T, Das SK, Fels DR, Hansen KS, Wong TZ, Dewhirst MW (2013). PET with (62)Cu-ATSM and (62)Cu-PTSM is a useful imaging tool for hypoxia and perfusion in pulmonary lesions. AJR Am J Roentgenol.

[15] Zegers CM, van Elmpt W, Szardenings K, Kolb H, Waxman A, Subramaniam RM (2015). Repeatability of hypoxia PET imaging using [F]HX4 in lung and head and neck cancer patients: a prospective multicenter trial. Eur J Nucl Med Mol Imaging.

[16] Klaassen R, Bennink RJ, van Tienhoven G, Bijlsma MF, Besselink MG, van Berge Henegouwen MI (2015). Feasibility and repeatability of PET with the hypoxia tracer [F]HX4 in oesophageal and pancreatic cancer. Radiother Oncol.

[17] Peeters SG, Zegers CM, Lieuwes NG, van Elmpt W, Eriksson J, van Dongen GA (2015). A comparative study of the hypoxia PET tracers [(1)(8)F]HX4, [(1)(8)F]FAZA, and [(1)(8)F]FMISO in a preclinical tumor model. Int J Radiat Oncol Biol Phys.

[18] Dubois LJ, Lieuwes NG, Janssen MH, Peeters WJ, Windhorst AD, Walsh JC (2011). Preclinical evaluation and validation of [18F]HX4, a promising hypoxia marker for PET imaging. Proc Natl Acad Sci U S A.

[19] Peeters SG, Zegers CM, Biemans R, Lieuwes NG, van Stiphout RG, Yaromina A (2015). TH-302 in combination with radiotherapy enhances the therapeutic outcome and is associated with pretreatment [18F]HX4 hypoxia PET imaging. Clin Cancer Res.

[20] Reischl G, Dorow DS, Cullinane C, Katsifis A, Roselt P, Binns D, et al. Imaging of tumor hypoxia with [124I]IAZA in comparison with [18F]FMISO and [18F] FAZA—first small animal PET results. J Pharm Pharm Sci. 2007;10:203–11. 17706178

[21] Valtorta S, Belloli S, Sanvito F, Masiello V, Di Grigoli G, Monterisi C (2013). Comparison of 18F-fluoroazomycin-arabinofuranoside and 64Cu-diacetyl-bis(N4-methylthiosemicarbazone) in preclinical models of cancer. J Nucl Med.

[22] Chen W, Delaloye S, Silverman DH, Geist C, Czernin J, Sayre J, et al. Predicting treatment response of malignant gliomas to bevacizumab and irinotecan by imaging proliferation with [18F]fluorothymidine positron emission tomography: a pilot study. J Clin Oncol. 2007;25:4714–21. doi:10.1200/JCO.2006.10.5825. 10.1200/JCO.2006.10.5825 17947718

[23] Mudd SR, Holich KD, Voorbach MJ, Cole TB, Reuter DR, Tapang P (2012). Pharmacodynamic evaluation of irinotecan therapy by FDG and FLT PET/CT imaging in a colorectal cancer xenograft model. Mol Imaging Biol.

[24] Kalra AV, Kim J, Klinz SG, Paz N, Cain J, Drummond DC (2014). Preclinical activity of nanoliposomal irinotecan is governed by tumor deposition and intratumor prodrug conversion. Cancer Res.

[25] Kang MH, Wang J, Makena MR, Lee JS, Paz N, Hall CP (2015). Activity of MM-398, nanoliposomal irinotecan (nal-IRI), in Ewing’s family tumor xenografts is associated with high exposure of tumor to drug and high SLFN11 expression. Clin Cancer Res.

[26] Von Hoff D, Li C, Wang-Gillam A, Bodoky G, Dean A, Jameson G (2014). NAPOLI-1: randomized phase 3 study of MM-398 (nal-IRI), with or without 5-fluorouracil and leucovorin, versus 5-fluorouracil and leucovorin, in metastatic pancreatic cancer progressed on or following gemcitabine-based therapy. Ann Oncol.

[27] Feig C, Gopinathan A, Neesse A, Chan DS, Cook N, Tuveson DA (2012). The pancreas cancer microenvironment. Clin Cancer Res.

[28] Baker JH, Lam J, Kyle AH, Sy J, Oliver T, Co SJ (2008). Irinophore C, a novel nanoformulation of irinotecan, alters tumor vascular function and enhances the distribution of 5-fluorouracil and doxorubicin. Clin Cancer Res.

[29] Sadanandam A, Lyssiotis CA, Homicsko K, Collisson EA, Gibb WJ, Wullschleger S (2013). A colorectal cancer classification system that associates cellular phenotype and responses to therapy. Nat Med.

[30] Busk M, Munk OL, Jakobsen S, Wang T, Skals M, Steiniche T (2010). Assessing hypoxia in animal tumor models based on pharmocokinetic analysis of dynamic FAZA PET. Acta Oncol.

[31] Mortensen LS, Johansen J, Kallehauge J, Primdahl H, Busk M, Lassen P (2012). FAZA PET/CT hypoxia imaging in patients with squamous cell carcinoma of the head and neck treated with radiotherapy: results from the DAHANCA 24 trial. Radiother Oncol.

[32] Vines D, McKee T, Mahmood J, Keller H, Jaffray D (2013). Reproducibility of 18F-FAZA PET-CT mouse imaging. J Nucl Med.

[33] Pommier Y (2006). Topoisomerase I, inhibitors: camptothecins and beyond. Nat Rev Cancer.

[34] Stylianopoulos T, Martin JD, Snuderl M, Mpekris F, Jain SR, Jain RK (2013). Coevolution of solid stress and interstitial fluid pressure in tumors during progression: implications for vascular collapse. Cancer Res.

[35] Chen PY, Ozawa T, Drummond DC, Kalra A, Fitzgerald JB, Kirpotin DB (2012). Comparing routes of delivery for nanoliposomal irinotecan shows superior anti-tumor activity of local administration in treating intracranial glioblastoma xenografts. Neuro Oncol.

[36] Roy AC, Park SR, Cunningham D, Kang YK, Chao Y, Chen LT (2013). A randomized phase II study of PEP02 (MM-398), irinotecan or docetaxel as a second-line therapy in patients with locally advanced or metastatic gastric or gastro-oesophageal junction adenocarcinoma. Ann Oncol.

