# Supplementary material for: Longitudinal tumor hypoxia imaging with [18F]FAZA-PET provides early prediction of nanoliposomal irinotecan (nal-IRI) treatment activity
Source: EJNMMI Res. 2015 Oct 19;5:57. doi: 10.1186/s13550-015-0135-x (PMC4610963; doi:10.1186/s13550-015-0135-x)

Suppl. Figure S1. Mean FAZA administration dose (MBq/g) calculated for animals belonging to each treatment group over 7 imaging sessions.

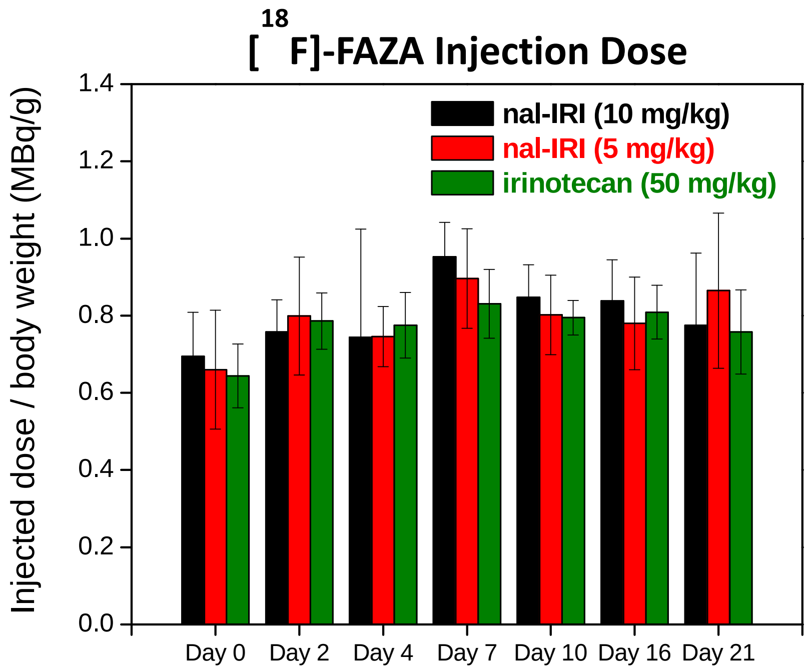

Suppl. Figure S2. Tumor volume correlation between measurements performed using caliper and CT.

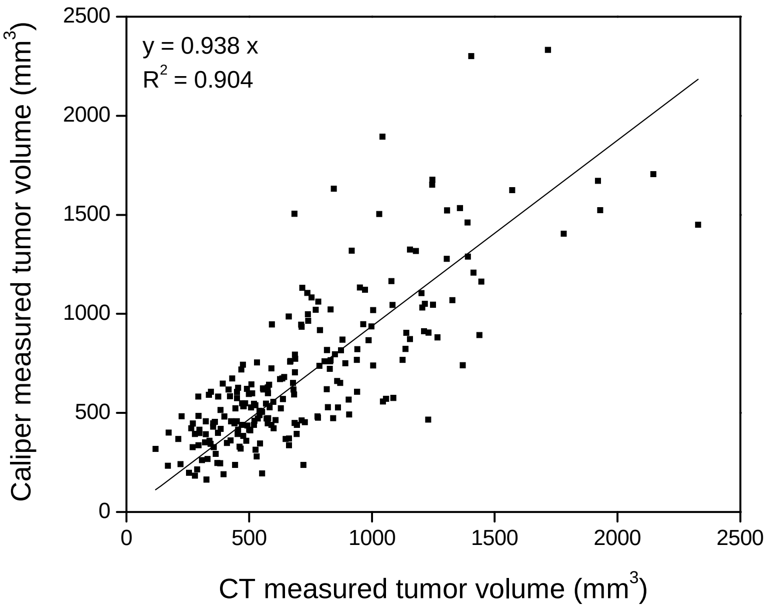

Suppl. Figure S3. Longitudinal muscle FAZA uptake (%ID/g) measured from the CT data set over a 21-day period following the first treatment dose administration (day 0). Each data point (except for day 21, transparently masked in grey) represents the mean ± standard deviation obtained from 5 animals.

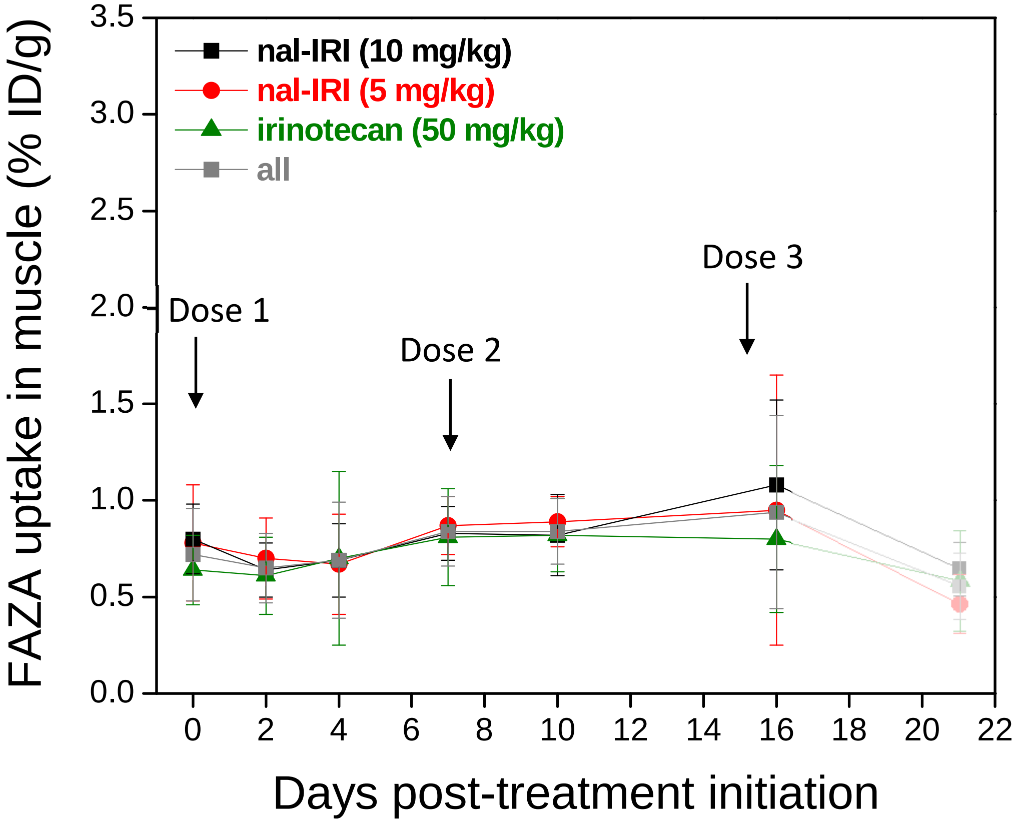

Supplement: Additional file 1: — Figures S1–S3. Figure S1. Mean FAZA administration dose (MBq/g) calculated for animals belonging to each treatment group over 7 imaging sessions. Figure S2. Tumor volume correlation between measurements performed using caliper and CT. Figure S3. Longitudinal muscle FAZA uptake (%ID/g) measured from the CT data set over a 21-day period following the first treatment dose administration (day 0). Each data point (except for day 21, transparently masked in gray) represents the mean ± standard deviation obtained from 5 animals. (PDF 86 kb) [file 13550_2015_135_MOESM1_ESM.pdf]
